# Benefits and Barriers of Caregiver App Engagement for Supporting Diverse Children With Asthma: Mixed Methods Study

**DOI:** 10.2196/69755

**Published:** 2025-12-04

**Authors:** Kandia Lewis, Cynthia M Zettler-Greeley, Amy Milkes, Kathryn V Blake

**Affiliations:** 1 Virtual Health and Informatics, Nemours Children’s Health, Wells Fargo Tower, 8th Floor, 1600 Rockland Road, Wilmington, DE, 19803, United States, 1 302-298-7779; 2 Virtual Health and Informatics, Nemours Children’s Health, Jacksonville, FL, United States; 3 Center for Healthcare Delivery Science, Nemours Children’s Health, Jacksonville, FL, United States; 4 Center for Pharmacogenomics and Translational Research, Nemours Children’s Health, Jacksonville, FL, United States

**Keywords:** digital health, pediatric asthma, app engagement, health care, education

## Abstract

**Background:**

Asthma is one of the most common pediatric conditions affecting millions of US children. Digital health apps may provide children and their caregivers (parents or legal guardians) with ways to manage asthma and improve health and educational outcomes.

**Objective:**

As digital health technology becomes more prevalent to help manage chronic conditions, like asthma, this study examined the reported benefits and barriers of caregiver interactions with an asthma-specific app. The app, created by physicians and digital health development professionals, was designed to educate, inform, and help caregivers manage the health of their child. We evaluated app logins and feature use (collectively defined as “app engagement”) for caregivers of children with asthma aged 5‐11 years. We examined whether (1) app engagement differed due to children’s demographic and asthma health characteristics, (2) themes about app engagement emerged from caregiver-reported app experiences, (3) these themes correlated with demographic and asthma health characteristics, and (4) engagement with the app was associated with reduced school absences.

**Methods:**

Eighty caregivers and their children with asthma participated between September 2019 and November 2020. Pretest (Time 1) and posttest (Time 2) data were collected over 6 months on caregiver and child demographic and health characteristics, health care usage, app engagement, and app experiences. Additionally, caregiver app engagement data and child health care data were collected retrospectively, 2 years prior to the start of the study. We used a mixed methods design, which included correlation, regression, chi-square, and content analysis to examine caregiver app engagement.

**Results:**

Most caregivers were mothers (76/80, 95%) and had a college degree (45/80, 56%). Children’s mean age was 8.76 (SD 1.79), and all were English speakers (80/80, 100%). About half of the participants were White children (43/80, 54%), and 26% (21/80) of them had uncontrolled asthma. Logistic regression revealed that caregivers of White children (OR [odds ratio] 8.57, 95% CI 1.68-43.65) with uncontrolled asthma (OR 17.81, 95% CI 2.36-134.24) who earned a college degree (OR 6.94, 95% CI 1.38-34.87) were statistically significantly more likely to use the app than caregivers of children of other races with controlled asthma without a college degree (*P*<.001). Qualitative findings support and expand on the logistic regression results. Five themes regarding app engagement emerged, including relevancy, acceptability and understandability, technology limitations, educational barriers, and information and communication benefits. Caregivers also identified specific app features that may promote child health and education.

**Conclusions:**

Understanding caregiver and child experiences in using digital health technologies for managing asthma may inform ways to support app engagement among caregivers and their children in the effort to improve patient health outcomes.

## Introduction

### Current Knowledge of Asthma and Digital Health

Childhood asthma is a chronic lung disease that affects 4.7 million children in the United States [1]. Uncontrolled asthma can be disruptive and costly to families and payors. Approximately 60% of children with asthma between 5 and 11 years of age have an asthma exacerbation each year, resulting in school absences (52%), emergency department visits (19%), and hospitalizations (5%) [2]. Uncontrolled asthma also contributes to an annual economic burden on families and health care systems. In 2013, the estimated total direct costs of pediatric asthma in the United States approached 6 billion dollars, averaging between US $3076 and US $13,612 per child [3]. These health and economic burdens disproportionately affect children who are Black, Latinx, and from lower-income families, due in part to systemic racism and unequal access to quality health care, housing, and education [4,5].

Compounding the health and economic issues, children with asthma are at higher risk for educational difficulties stemming from chronic absenteeism [6]; asthma exacerbations contribute to about 10.5 million school absences each year [7]. As chronic absenteeism contributes to lower math and reading abilities compared to children who are not repeatedly absent [8], there is a need to develop tools to improve children’s asthma to keep them healthy and in the classroom. One worthwhile approach to support improved asthma management may be to use digital health apps [9]. However, because a digital divide in usage exists based on race, ethnicity, income, and geographic location [10-14], the question remains as to whether digital health tools are appropriate for use across a diverse population of caregivers (parents or legal guardians) and their children.

### Digital Health Apps: How to Support Asthma Management?

Leveraging digital health apps to improve pediatric asthma management may contribute to improved asthma health for children from underserved populations and those with uncontrolled asthma. Recent indications show digital health technologies may aid in addressing health equity concerns among marginalized patient populations as they frequently engage with specific app features [14]. Moreover, digital health tools are more likely to be used when children have uncontrolled asthma [9,10], as they often require frequent health care usage and engage with several digital health tools [14]. These tools may help improve children’s health outcomes [15], as studies reveal these devices support medication adherence [16] and caregiver asthma knowledge [17], contributing to improved pediatric asthma health within 6 months [15,18]. As these patient populations have recently increased usage of mobile devices [19], including engagement with digital health apps [20], there is an opportunity to increase their access to health care and improve their health outcomes. Moreover, improved asthma health may reduce school absenteeism, thereby improving educational outcomes [21].

However, the reasons for app engagement are unclear, with studies highlighting potential barriers to app engagement; thus, further study is warranted. Access issues may include a lack of broadband internet or smartphone devices [22]. However, many individuals who have access to these technologies have suboptimal engagement due to a lack of knowledge, understanding, or guidance on how or why to use them for managing health needs [12]. Further, specific features may be more relevant for certain populations. For example, one study found that families receiving Medicaid—US government-funded health care services for low-income patients—accessed digital health educational resources more often than non-Medicaid recipients [14]. The authors posited that the frequent use of educational features among Medicaid recipients was due to a lack of access to health care information elsewhere. Further research is needed to understand these apparent contradictions in app use across patient populations.

Benefits and barriers of app engagement include whether patients consider the app relevant, understandable, and acceptable to use [23-25]. Moreover, as certain features may be considered more beneficial for certain populations [14], benefits and barriers may differ across diverse patient populations. As digital health tools become more widely implemented in pediatric health care institutions, it is vital to examine whether these technologies may yield equitable benefits for children. Consequently, it would be valuable to learn whether and why caregivers choose to use health apps for managing their child’s health care. In addition to supporting health outcomes, this information could assist app developers in targeting users’ needs, interests, and preferences, potentially motivating increased app engagement.

### Purpose

The purpose of this study was to examine the benefits and barriers of digital health app engagement among caregivers of school-aged children with asthma. As such, we focused on children aged 5‐11 years because after the age of 11 years, many children take responsibility for most of their asthma care [26]. Understanding factors that contribute to caregiver app engagement may help to uncover whether app engagement correlates with improved child health and education, including for those at risk for these poor outcomes. We explored four research questions:

1. Does app engagement differ based on demographic and asthma health characteristics? We hypothesized that (1) caregivers of White children and caregivers with higher socioeconomic status would be more likely to engage with the app than caregivers of children of other races and lower socioeconomic status [11,14], and (2) caregivers of children with uncontrolled asthma would be more likely to engage with the app than caregivers of children with controlled asthma [9,10,14,15].
2. Do app engagement themes emerge from caregiver-reported experiences about the app? We hypothesized that caregiver-reported experiences about app engagement would yield themes around relevancy, acceptability, and understandability [23-25].
3. Do app engagement themes correlate with demographic and asthma health characteristics? We wanted to understand whether identified themes related to benefits and barriers of app engagement vary across diverse children with asthma. We hypothesized that (1) families with higher educational attainment would endorse more benefits of app engagement than less educated families [11,12] and (2) those of children with uncontrolled asthma would endorse more benefits of app engagement than families of children with controlled asthma [9,10,14,15].
4. Is app engagement related to reduced school absences? We hypothesized app engagement would help improve asthma management [15,18], thereby reducing chronic absenteeism.

## Methods

### Study Design and Procedure

This mixed methods, multisite, observational retrospective and prospective study included 6 months of active data collection and 2 years of retrospective data collection. Retrospective asthma health data were collected to avoid potential underreporting, as seasonality contributes to asthma management [27]. Moreover, obtaining 2 years of retrospective asthma control scores increased the likelihood of their collection, which are provided by caregivers and entered into the electronic health record (EHR) at scheduled patient care visits. Retrospective app engagement data, commensurate with asthma health data, and prospective data were recorded in the app. Prospective data were collected at study start (Time 1) and 6 months later (Time 2), consistent with previous digital health and asthma research [15,18].

### Participants

Caregivers of children who were patients of Nemours Children’s Health (NCH) and who had an asthma diagnosis were emailed recruitment notifications with access to a secure REDCap (Research Electronic Data Capture; Vanderbilt University) link to learn more about the study. Of the 154 caregivers who accessed the link, 148 (96%) were interested, and 126 (85%) of those interested were eligible for enrollment. Children were eligible if they were aged 5‐11 years, had a diagnosis of asthma, and had a visit with a provider in the Nemours allergy or pulmonology departments within a year prior to consent. Caregivers were required to be a parent or legal guardian fluent in conversational English to match the language of the app. Families across Nemours US locations (Northeast and Southeast) were eligible whether they had the app or not. Per consent, we limited enrollment to 1 child per family. Almost two-thirds of eligible participants (64%, 80/126) consented to participate, 100% (80/80) completed all pretest study activities, and 80% (64/80) completed both pretest and posttest study activities.

### Measures and Data Collection

#### Demographic Questionnaire

The Parent Research Questionnaire was a 28-item researcher-developed questionnaire about demographic characteristics (eg, income, race, ethnicity, and education level) and health characteristics (eg, family history of asthma). The questionnaire took about 10 minutes to complete. It has been used in previously published work [9], and it has asthma-specific items that were developed for study purposes, such as age of asthma diagnosis, family history of asthma, and asthma triggers. Data were collected at Time 1.

#### App Experience Survey

The App Experience survey was a 36-item researcher-developed questionnaire about caregiver app engagement (Section A in Multimedia Appendix 1) to understand caregiver engagement of a novel digital health app, which has been used in previously published work [9]. The survey took about 15 minutes to complete and included multiple-choice and open-ended items about feature engagement and app experiences (eg, reasons for using or not using the app, frequency of features used, and benefits and barriers of app use) and school absences due to asthma. Data were collected at Time 1 and Time 2.

#### Nemours App

Physicians and digital health development professionals at NCH developed a caregiver-facing digital health app, the “Nemours app,” with features designed to support patients at NCH with asthma and their caregivers. Caregivers log in to the app via mobile devices or desktops and are logged out after 30 minutes of inactivity. The app has been in use since February 2018 and includes traditional portal mainstays, such as telehealth and messages, which can be used as frequently as desired, as well as asthma-specific features (Table 1). As this was an observational study, only resources provided by NCH were available, including contact information for technical support, an app demonstration video, and medication reminders (if selected by caregivers). Participants reported hearing about the app via emails, providers, family, friends, searches for digital health apps, and this study.

We defined “app engagement” as logging into the app at least once and using at least 1 identified app feature (Table 1). We defined a caregiver who did not log in or use any features during the study as a “nonapp engager” [28].

**Table 1.** App features.

Feature	Type	Description
Reminders	Asthma-specific	Set medication reminder notifications to support asthma medication adherence.
Medication images	Asthma-specific	View images to identify controller versus quick relief medications.
Medication videos	Asthma-specific	Watch videos demonstrating proper asthma medication usage.
Asthma educational resource	Asthma-specific	Read articles and terminology word banks to learn about asthma.
Asthma symptom tracker	Asthma-specific	Track asthma control levels, symptoms, limitations, and the air quality index. The data are linked to the electronic health care record so that providers may access patients’ tracker entries between office visits.
Asthma action plan	Asthma-specific	Access individualized, provider-created, asthma action plans. Download and share it as a PDF or use it interactively to receive real-time feedback on actions that may help to resolve an exacerbation.
Air quality alerts	Asthma-specific	Receive alerts for dangerous levels of air quality to remind children to carry quick relief medication.
Telehealth	General	Participate in video visits with health care providers.
Messages	General	Communicate with health care providers.

#### Health Care Records

Data were obtained for asthma diagnosis and severity (as specified by the child’s provider), asthma control, and health care usage (eg, asthma-related emergency department visits, urgent care visits, and hospitalizations) from the EHR. Asthma control was obtained from childhood asthma control test (C-ACT) scores in the EHR. Available C-ACT scores ranged from 0 to 9. Mean scores provided an average asthma control level per child. Scores above 19 were defined as “controlled,” and scores of 19 or below were defined as “uncontrolled” [29].

### Power Analysis

This study was part of a larger project for which we conducted an a priori power analysis to determine the sample size [9]. Sufficient power was estimated with n=40. To account for the 50% attrition typical in digital health research [30], we enrolled 80 caregivers and their children.

### Data Analysis

We used a mixed methods approach to examine caregiver app engagement. We first conducted a preliminary correlation analysis to identify statistically significant (*P*<.05) demographic (eg, child age, government public assistance, parent learning problems, and transportation difficulties) and health characteristics (eg, family asthma history, asthma triggers, and health care usage) to include as predictors in the analysis for research question 1.

To address the first research question, we conducted logistic regression to evaluate the relation between the identified demographic and health characteristics and caregiver app engagement. Child race, maternal education level, and asthma control level were statistically significantly related to app engagement and were used as predictors in the regression model. The variables were dummy coded for the analysis: maternal education level (0=high school degree or less, 1=associate’s, bachelor’s degree or higher), child race (0=Other races, 1=White child), and asthma control level (0=controlled, 1=uncontrolled). Logistic regression was used because the outcome variable, app engagement, was dichotomous (0=nonapp engagement, 1=app engagement) [31]. Listwise deletion was used to address missing values for asthma control level in the analysis. As a result, 14 nonapp engagers and 13 app engagers were removed from the final regression analysis (n=53). For research question 1, missing data ranged between 0% and 34% (asthma control level missing at 27/80, 34%) and were missing completely at random per Little Missing Completely at Random test (*χ*^*2*^_3_=3.5; *P*=.33). Multiple imputation analyses were similar to the nonmultiply imputed analyses; thus, nonmultiply imputed findings are presented. For transparency, we included the multiply imputed findings (Section B in Multimedia Appendix 1).

We also conducted post hoc analyses because although all participants completed the Time 1 app experiences survey, 19% (15/80) of the data were missing for the Time 2 survey. Thus, we examined whether there was a difference between app engagers and nonapp engagers who completed the Time 2 survey. We also examined the relation between key demographic and health characteristics and completion of the Time 2 survey. Further, we examined whether frequency of app use was correlated with caregiver-child demographic and health characteristics.

For the second research question, content analysis was conducted to identify app engagement themes about caregiver app experiences. Caregiver responses were organized and coded in Excel (Microsoft) by the first author. Analysis of codes and themes was conducted in SPSS (IBM Corp). We used a combined approach. First, we used a deductive approach to examine a priori codes that were created to uncover themes regarding app benefits and barriers, including relevancy, understandability, and acceptability [23-25]. After that, we used an emergent approach because of the novelty of the Nemours app by using open coding in which additional codes could be added after initially reviewing the data [32]. We also used axial coding to combine related codes [33]. Coding was considered complete when only a few uncoded data remained [34,35]. Finally, the first and third authors double-coded 31% (5/16) of randomly selected caregiver open-ended response sections, which demonstrated strong percent agreement (0.92) and high interrater reliability (κ=0.87). For the third research question, we used nonparametric correlation analyses (Spearman) due to nonnormality to examine the extent to which themes about app relevancy, understandability, acceptability, benefits, and barriers were related to demographic and asthma health characteristics.

To address the fourth research question, we used chi-square analysis because both variables, app engagement (0=nonapp engagement, 1=app engagement), and school absences (0=none, 1=1‐5, 2=6‐10, 3=more than 10), were categorical. We examined whether app engagement was related to decreased school absences between Time 1 and Time 2. We calculated the difference score between school absences at Time 1 and Time 2. Initially, listwise deletion was used, resulting in 13 nonapp engagers and 3 app engagers being removed from the analysis (n=64). Overall, missing data ranged between 0% and 20% (school absences missing at 16/80, 20%). Data were missing not completely at random per Little Missing Completely at Random test (*χ*^2^_1_=15.0; *P*<.001), as nonapp engagers had more missing school absence data (13/31, 42%) than app engagers (3/49, 6%). As multiple imputation typically leads to less biased results than listwise deletion [36,37], we conducted these analyses and included predictor and covariate variables to help account for missingness [36]. The multiply imputed analyses yielded significant results, whereas the nonmultiply imputed analyses did not. As such, we present the nonmultiply imputed findings because it is likely a more conservative estimate. For transparency, we also included the multiply imputed findings (Section C in Multimedia Appendix 1). Additionally, we conducted post hoc analyses because, given that maternal education, race, and asthma control are associated with school absences [38], we examined whether these characteristics, plus app engagement, were related to a decline in school absences.

### Ethical Considerations

The study was conducted between September 2019 and November 2020 and was approved by the Nemours Institutional Review Board (approval number 1422315). REDCap, a web-based app for secure data capture and storage [39,40], was used for study recruitment (via a rolling admission process), parental or legal guardian consent, data collection, and participant communication. Survey data were collected securely online via REDCap and items appeared in a consistent order. Page numbers varied from 4 to 7 because an adaptive format was used, and items ranged between 8 and 14 per page because of the variation in item format. Participants were informed of the study investigators, purpose, approximate time to complete surveys, security of data storage on password-protected files or servers, and the voluntary nature of participation. Quantitative data were obtained from multiple-choice survey items and qualitative data were obtained from open-ended survey items. A US $50 gift card was provided to incentivize participation. For additional information, see Lewis et al [9].

## Results

### Participant Characteristics

Participating children’s race and ethnicity largely reflected that of pediatric patients with asthma in the US population, including a slight overrepresentation of White children and a slight underrepresentation of Latinx children [1]. The child sample, as identified by caregivers, and the US population, respectively, was 54% (43/80) and 44% (2,074,381/4,675,475) White, 24% (19/80) and 23% (1,058,310/4,675,475) Black, 12% (10/80) and 5% (221,110/4,675,475) multiracial, 1% (1/80) and 2% (108,819/4,675,475) Asian, and 9% (7/80) unreported; 12% of the child sample (10/80) and 24% (1,102,471/4,675,475) of the US population were Latinx. The 49 caregivers who engaged with the app logged in at least 3 times, exceeding our engagement criteria (at least one log in), and engaged with at least one of these features, including reminder notification (n=1, 2%), medication images (n=17, 35%), asthma symptom tracker (n=32, 65%), asthma action plan (n=11, 22%), air quality index (n=9, 18%), telehealth video visits (n=9, 18%), and messages (n=28, 57%). Child and caregiver demographic statistics are presented in Table 2. For additional information about participants, see Lewis et al [9].

**Table 2.** Child and caregiver demographic statistics by app use.

Characteristic	All (N=80)		Engage (n=49)		Nonengage (n=31)	
Child age, mean (SD)	8.76 (1.79)		8.57 (1.79)		9.07 (1.77)	
Mean C-ACT^a^ score^b^ , mean (SD)	20.84 (3.81)		20.18 (4.27)		22.24 (2.10)	
English is primary language, n (%)	80 (100)		49 (100)		31 (100)	
Child race, n (%)						
White	43 (54)		32 (69)		11 (41)	
Black	19 (24)		9 (20)		10 (37)	
Multiracial	10 (12)		4 (9)		6 (22)	
Asian	1 (1)		1 (2)		0 (0)	
Child ethnicity (Hispanic or Latinx), n (%)	10 (12)		4 (8)		6 (19)	
Grade at Time 1, n (%)						
Daycare–Kindergarten	23 (29)		13 (27)		10 (32)	
First–second	24 (30)		16 (33)		8 (26)	
Third–fourth	22 (27)		11 (22)		11 (35)	
Fifth–sixth	11 (14)		9 (18)		2 (6)	
Asthma control, n (%)						
Uncontrolled^b^	21 (26)		19 (39)		2 (6)	
Controlled^b^	32 (40)		17 (35)		15 (48)	
Asthma severity, n (%)						
Intermittent	14 (18)		12 (24)		2 (6)	
Persistent	66 (82)		37 (76)		29 (94)	
Allergies	62 (78)		39 (80)		23 (74)	
Asthma-related absences Time 1, n (%)						
None	28 (35)		20 (41)		8 (26)	
1-5 days	36 (45)		20 (41)		16 (51)	
More than 6 days	16 (20)		9 (18)		7 (23)	
Asthma-related absences Time 2, n (%)						
None^c^	33 (41)		24 (49)		9 (29)	
1-5 days^c^	23 (29)		17 (35)		6 (19)	
More than 6 days^c^	8 (10)		5 (10)		3 (10)	
Caregiver relationship to child, n (%)						
Mother	76 (95)		46 (94)		30 (97)	
Father	2 (3)		1 (2)		1 (3)	
Other (eg, grandparent)	2 (3)		2 (4)		0 (0)	
Maternal highest degree, n (%)						
High school diploma or GED^d^	14 (18)		5 (10)		9 (29)	
Some college or trade school	21 (26)		11 (22)		10 (32)	
Associate’s or bachelor’s degree	24 (30)		19 (39)		5 (16)	
Graduate or professional school	21 (26)		14 (29)		7 (23)	

a C-ACT: childhood asthma control test.

b Data for 27 children unreported.

c Data for 16 children unreported.

d GED: General Education Development.

### Predicting App Engagement From Demographic Survey and Health Care Records

The full logistic regression model was statistically significant, (*χ*^2^_3_=24.5; *P*<.001), and well specified, as the Hosmer-Lemeshow Test was not statistically significant (*P*=.84). The model explained approximately 52% of the variance for app engagement (Nagelkerke R^2^=0.52) and correctly classified 83% of the cases. Caregivers of White children were about 8.5 times more likely to engage with the app than caregivers of children of other races when controlling for maternal education and child asthma control level (OR [odds ratio] 8.57, 95% CI 1.68-43.65; *P*=.01). Caregivers with a college degree were nearly 7 times more likely to engage with the app than caregivers with a high school diploma when controlling for child race and asthma control level (OR 6.94, 95% CI 1.38-34.87; *P*=.02). Caregivers of children with uncontrolled asthma were almost 18 times more likely to engage with the app than caregivers of children with controlled asthma when controlling for maternal education and child race (OR 17.81, 95% CI 2.36-134.24; *P*=.01).

### Post Hoc Analyses to Examine Survey Completion and Frequency of App Use

Responses about app engagement were skewed to those who engaged with the app, as they were statistically significantly more likely to complete the Time 2 survey than those who did not engage with the app (*χ*^2^_1_=17.9; *P*<.001). However, logistic regression and crosstabulations revealed no statistically significant relation between key participant characteristics and Time 2 app experience survey completion. Moreover, frequent app engagement (logged in ≥24 times) was not significantly correlated with (1) child and caregiver demographic characteristics including age, race, ethnicity, education, receiving public assistance, and transportation issues and (2) child health, including age diagnosed with asthma, family asthma history, asthma triggers, health care usage, and asthma control.

### Themes That Emerged via the App Experience Survey

Nearly all caregivers (75/80, 94%) responded to at least one of the 16 open-ended response sections in the survey with an average of 28.31 (SD 17.58) responses per section. Five themes (2 a priori—relevancy and acceptability or understandability—and 3 emergent—technology limitations, educational barriers, and information and communication benefits—themes) from 408 coded comments resulted from the content analysis about caregiver app experiences (Table 3). One theme was whether the app was relevant for family use to manage asthma, with more caregivers stating the app was not relevant (eg, child’s asthma was controlled; 12/80, 15%) than relevant (1/80, 1%). Another theme identified the “acceptability” and “understandability” of the app. More caregivers noted the app was easy to use and understand (56/80, 70%) relative to those who noted it was difficult to use and understand (23/80, 29%). Two themes were specifically related to barriers. First, caregivers noted technology limitations (17/80, 21%), such as difficulties with internet connection and technical issues (eg, during telehealth). Educational barriers (29/80, 36%) included being (1) unaware of the app or its features, (2) unclear about the app’s potential benefits or purpose, and (3) unsure of how to use the app. The final theme pertained to the app’s information and communication benefits (51/80, 64%), such as gaining information about child health and being able to communicate with a child’s care team.

**Table 3.** App engagement themes.

Category (n=80)	Themes and examples	Caregivers, n	Total comments, n
Relevancy			
Benefits	App Engager: “I don’t think I was aware there was a newer app. [Her] asthma triggers have been a mystery so tracking her symptoms, good/bad days will be helpful.”	1	1
Barriers	App Engager: “Not sure how [the app] will help us. My daughter seems to be doing well.” Nonapp Engager: “She is inside more due to [COVID-19].” Nonapp Engager: “I don’t generally use the app. My providers haven’t really made it into a tool we need...”	12	14
Acceptability or understandability (ease of use or clarity)			
Benefits	App Engager: “I think this is very user friendly and everything in one place is very convenient.” App Engager: “On my phone and convenient.” App Engager: “It’s easy to navigate and very comprehensive.”	56	118
Barriers	App Engager: “Difficult to understand and use.” Nonapp Engager: “Difficult to find things quickly.”	23	43
Technology limitations			
Barriers	App Engager: “Lack of a strong internet connection.” Nonapp Engager: “Having to pay for internet services.” Nonapp Engager: “It has technical issue with video appointments.”	17	34
Educational			
Barriers	App Engager: “I have no idea what I am supposed to use the app for in regards to my child’s asthma.” App Engager: “Should be more education to parents about what we should be using the app for.” Nonapp Engager: “I wasn’t aware of the videos and fun facts about learning more about asthma.”	29	35
Information and communication			
Benefits	App Engager: “Love the ability to message providers directly.” App Engager: “A good way to get data and communicate with your provider.” App Engager: “Easily shows doctor current health.”	51	163

Content analysis also revealed specific app features caregivers identified as a benefit to app engagement based on their experience with the app or based on app feature information shared via the study survey (Table 4). The most frequently reported beneficial app features were messages (25/80, 31%), access to health care records (24/80, 30%), and the asthma symptom tracker (19/80, 24%). These app features were often noted as beneficial for communication purposes and for gathering and sharing information about children’s asthma health.

**Table 4.** Caregiver-reported beneficial app features.

Features (n=80)	Examples	Caregivers, n	Total comments, n
Air quality index	App Engager: “To check air Quality”	5	5
Asthma action plan	Nonapp Engager: “To print action plan for schools and camp”	6	6
Telehealth video visits	App Engager: “Having the option to stay home and still have a follow up visit with the doctor”	8	10
Medication reminders, refills, and education	App Engager: “I use it for action plans and prescription renewals.”	9	11
Asthma symptom tracker	App Engager: “I like the asthma tracker so that I can see if there is a pattern to when his asthma flares up.”	19	25
Health care records	App Engager: “I can see details from all past appointments. I find it easier to use than [our] pediatricians so I use it if I have a question about her growth, asthma and medication history” App Engager: “I like that I can refer back to this app after an appointment in case I forgot something the doctor said.”	24	32
Messages	App Engager: “Love the ability to message providers directly.”	25	39

### Correlation Between App Engagement Themes and Demographic and Health Characteristics

Statistically significant correlations were found between demographic and health characteristics and app engagement themes, specifically themes around relevancy, acceptability or understandability, educational barriers, and information and communication benefits (Table 5). Regarding relevancy, caregivers of children with dust allergies reported more benefits to understanding how the app could be helpful (ρ=0.23; *P*=.04), whereas caregivers with learning problems noted more barriers (ρ=0.27; *P*=.02). Regarding acceptability or understandability, caregivers of Asian children noted more benefits regarding the ease of use and clarity of the app (ρ=0.23; *P*=.04), whereas caregivers of children with pollen allergies (ρ=0.30; *P*=.01) and controlled asthma (ρ=−0.30; *P*=.03) shared more barriers. Caregivers of Asian children (ρ=0.28; *P*=.01) and of children with pollen allergies (ρ=0.24; *P*=.03) reported more app educational barriers. Finally, caregivers of White children (ρ=0.30; *P*=.01) and caregivers who did not receive public assistance (ρ=–0.37; P<.001) reported more access to asthma-related health information and provider communication benefits because of their app engagement.

**Table 5.** Significant Spearman correlations between app themes and demographic and health characteristics.

Characteristic	Race-Asian		Race-White		Public assistance		Learning problems		Dust		Pollen		Asthma control	
	Spearman rank correlation (ρ)	*P* value	Spearman rank correlation (ρ)	*P* value	Spearman rank correlation (ρ)	*P* value	Spearman rank correlation (ρ)	*P* value	Spearman rank correlation (ρ)	*P* value	Spearman rank correlation (ρ)	*P* value	Spearman rank correlation (ρ)	*P* value
Relevancy–benefits	–0.02	.85	0.10	.38	–0.07	.52	–0.03	.78	0.23^a^	.04	0.08	.47	0.17	.22
Relevancy–barriers	–0.08	.47	0.15	.18	–0.20	.08	0.27^a^	.02	0.05	.69	0.08	.47	–0.05	.72
Acceptability or understandability–benefits	0.23^a^	.04	0.13	.24	–0.15	.19	–0.02	.84	0.08	.50	0.12	.29	–0.08	.55
Acceptability or understandability-barriers	0.03	.79	0.18	.11	–0.02	.89	–0.06	.62	0.22	.05	0.30^a^	.01	–0.30^a^	.03
Education barriers	0.28^a^	.01	0.11	.36	–0.12	.29	–0.03	.78	0.06	.60	0.24^a^	.03	0.10	.52
Information and communication benefits	0.03	.79	0.30^a^	.01	–0.37^a^	<.001	0.03	.82	0.17	.13	0.18	.12	0.04	.79

a Indicates statistically significant correlations.

### Relation Between App Engagement and Caregiver-Reported School Absences

Chi-square analysis revealed that app engagement was not statistically significantly related to the school absence difference score (0=no change in absences, 1=absences reduced by 1 category, 2=absences reduced by 2 categories, 3=absences reduced by 3 categories) between Time 1 and Time 2 (*χ*^2^_3_=2.6; *P*=.46). As we did not find a statistically significant relation with this analysis, we also conducted a chi-square test to evaluate the difference in school absences for all children, regardless of app engagement. When examining this difference, we found a statistically significant decline in absences for all children between Time 1 and Time 2 (*χ*^2^_9_=99.9; *P*<.001). Further, we found that more caregivers reported “no absences” at Time 2 (33/80, 41%) than at Time 1 (28/80, 35%). We would expect fewer caregivers reporting “no absences” later in the year at Time 2, as we asked about cumulative school absences over the past year. These discrepancies suggest the results may be uninterpretable, warranting further research. We present this data, despite the limitations, because this was a planned study analysis. Additionally, as findings differed between nonmultiply imputed and multiply imputed data (Section C in Multimedia Appendix 1), the results must be interpreted with caution. Despite the discrepancies with the absentee data, for transparency, we included post hoc findings indicating child race and asthma control are statistically significantly related to school absences (Section D in Multimedia Appendix 1).

## Discussion

### Principal Findings

Understanding facilitators and challenges from caregivers and children who use digital health technology is key to uncovering ways to improve app engagement to potentially improve child health and educational outcomes. Using a mixed methods approach, this study demonstrated key demographic and health characteristics related to app engagement and revealed themes that further supported these findings. These themes may uncover factors that improve engagement among caregivers and children with diverse demographic and health characteristics. Three important findings were gleaned from this work, contributing to the nascent field of digital health research. First, quantitative and qualitative findings revealed racial, educational, and health-related differences in app engagement. Second, the app may have the potential to help improve communication between caregivers, children, and health care providers. Finally, caregivers identified app features that may help support child health and education.

### Gaps in App Engagement

Caregivers of White children with uncontrolled asthma who earned a college degree were more likely to engage with the app than caregivers of children of other races with controlled asthma without a college degree. This suggests there may be an educational and racial gap in app engagement, consistent with previous research [12-14]. As a digital divide persists [9-14], care must be taken to understand potential reasons for this gap in app engagement. In this study, caregiver-reported app experiences supported the quantitative results and provided potential reasons for a divide between families of different racial and educational backgrounds.

Regarding educational and racial gaps, caregivers with a history of learning problems in school and caregivers of Asian children reported more educational barriers to app engagement. These caregivers reported receiving limited information about how to use the app to help manage their child’s asthma or about the potential benefits of app engagement. This lack of education about the purpose and potential benefits of the app was well captured by this app engager’s response: “I have no idea what I am supposed to use the app for in regards to my child’s asthma.” Video tutorials may be useful in addressing this educational barrier by facilitating app engagement among caregivers and children [23], whereby potential benefits of app engagement are highlighted, and app feature use is demonstrated. Video tutorials could also identify available features, such as access to health care educational resources, which may enhance interest among families who may perceive or experience a lack of access to these resources [14]. Additionally, creating digital health video tutorials may be particularly beneficial for families from low educational backgrounds because video tutorials are supportive of families with low literacy skills, thus reducing educational and literacy-related barriers to understanding how and why to use digital health apps [17,41]. Thus, integration of more educational supports, such as video tutorials, may improve engagement by making app engagement easier, potentially narrowing health disparities among racially, ethnically, and educationally diverse populations [12].

Findings from this study, consistent with our previous study [9], revealed that caregivers of children with uncontrolled asthma were more likely to use the app than caregivers of children whose asthma was controlled. Caregivers of children with controlled asthma shared more barriers, with understanding and using the app with comments such as, “Not sure how [the app] will help us. My daughter seems to be doing well,” noting that the app is “Difficult to understand and use…” and “Difficult to find things quickly.” These caregivers may be unsure why they should use it (“Not sure how [the app] will help us...”). Perhaps video tutorials could also be used to inform families of the potential benefits of continued monitoring of asthma symptoms, even when asthma appears under control, given that asthma can be unpredictable and seasonal [27]. Additionally, caregivers of children whose asthma is controlled may be less familiar with navigating the app because they do not engage with the app, or they engage with it less frequently than caregivers of children whose asthma is uncontrolled. This suggests a barrier to app use may be due to a lack of instruction, further suggesting an app video tutorial may be beneficial. Others have found that asthma interventions appear to be more effective for children with uncontrolled asthma, as these children are most at risk for poor health outcomes [15]. As such, a 2-pronged approach may be a worthwhile solution, in which informational video tutorials provide potential benefits of app engagement for all children with asthma and also highlight features that may best serve children with uncontrolled asthma, including enhanced communication opportunities with their child’s asthma health care team.

### App-Facilitated Communication

Pediatric asthma management may be facilitated by improved communication between health care providers and families. When providers and families engage in effective communication strategies, clinical decisions can be made jointly, thereby assisting treatment plan adherence [42]. Notably, using digital health technology is associated with improved communication between families and providers [9,43]. A randomized controlled trial found that children who received remote patient monitoring and health care provider feedback demonstrated statistically significantly higher asthma treatment adherence than children who only received remote patient monitoring [44]. Consequently, communication with the health care team in conjunction with digital health technology may be key for effectively improving treatment plan adherence. Similarly, results from our previous study indicated that 67% (33/49) of caregivers who used a digital health app reported that using the app led to improved communication with their child’s health care team [9]. The qualitative results from this study further support this previous finding, as the most frequently reported app theme that emerged was about communication benefits. Specifically, 40% (163/408) of the comments referred to communication and information sharing as a benefit of app engagement. One app engager captured their experience with app-related communication by reporting it is, “A good way to get data and communicate with your provider.” As such, this study highlights that app-facilitated communication may serve to strengthen provider-family communication, which could help improve children’s asthma health.

Two app features frequently reported as helpful and that could promote communication were the message system and the asthma symptom tracker. When reporting about messages, an app engager shared they “Love the ability to message providers directly.” Another app engager noted the tracker is beneficial because they “…can see if there is a pattern to when [their child’s] asthma flares up.” These reports about app-facilitated communication features highlight tools that may support treatment adherence and improve asthma outcomes.

### Potential Benefits of the App for Health Care and Education

In addition to app-facilitated communication features, messages, and asthma symptom tracker, caregivers identified several other app features as potentially beneficial. Unsurprisingly, the 3 most frequently reported beneficial app features, messages, health care records, and the asthma symptom tracker, aligned with the most prominent theme about app information and communication benefits. As noted, caregivers shared that engaging with messages and the asthma symptom tracker supported communication between themselves and their child’s health care team. Caregivers also reported that having access to their child’s health care records was informative. For instance, one app engager noted, “I like that I can refer back to this app after an appointment in case I forgot something the doctor said.” Other features were also identified as informative, as caregivers reported that the air quality index helped them check the air quality, and app medication features could help with medication adherence. These features may help support asthma management and lead to improved health outcomes.

Despite the ambiguous school absence findings, many caregivers identified potential benefits of app features for school purposes, namely asthma-specific app features. For example, one non-app engager shared that they would “print action plan for schools and camp.” Sharing asthma treatment plans, particularly asthma action plans, with schools is recommended by the National Heart, Lung, and Blood Institute to inform school personnel of child-related asthma needs and keep children safe in schools and may help reduce school absences or time away from the classroom [6]. We also found an overall reduction in school absences from Time 1 to Time 2. However, future research is necessary to explain the overall reduction in school absences. This study took place during the early period of the COVID-19 pandemic, during which childhood asthma morbidity was significantly reduced [45], as children were inside more frequently and perhaps exposed to fewer triggers. This explanation was supported by findings from this study, as one caregiver shared that the reason they were not engaging with the app was because “She is inside more due to [COVID-19].” Despite the historical and school absentee data limitations, most caregivers reported the benefits of app engagement and highlighted asthma-specific features that may be beneficial for their child’s asthma-related health and education. As such, it is worthwhile for health care systems to consider that app engagement, particularly engagement with disease-specific app features, may be beneficial for the children and caregivers they serve.

### Limitations and Future Research

As many people were using virtual technologies for health care and for schooling during the study time frame (from September 2019 to November 2020) due to the COVID-19 pandemic, this may have influenced caregivers’ perspectives about engaging with digital health technology. Missing data were another limitation, as 20% (16/80) of school absentee data were missing at Time 2 and these data were not missing at random, likely because statistically significantly fewer nonapp engagers than app engagers completed the Time 2 survey. Post hoc analyses are consistent with previous research indicating that other characteristics, such as race and asthma control, are related to school absences [38]. However, we caution that the main and post hoc findings about school absences may be uninterpretable due to the history-related artifacts in the absentee data. Despite this limitation, we presented the data because assessing school absences was an important hypothesis to examine and part of the planned study analysis. Future digital health research is needed to examine the relationship among app engagement, absenteeism, and family characteristics to identify approaches that may help improve school attendance. A future study should also collect absentee data from school systems instead of relying on caregiver-reported data, which may help to limit any misreporting of the number of school days missed. For this observational study, we relied on existing data, using C-ACT data available in the EHR. Consequently, only a subsample of C-ACT scores was included in the analyses (53/80, 66%). Future research should collect these data directly for study purposes to limit the amount of missing data. Several biases must also be considered, as our findings may not generalize to a broader population. Although this study took place across the northeastern and southeastern United States, we examined a digital health app designed to support pediatric patients at a specific health care institution and developed a specific survey to obtain data about caregivers’ app experience. As such, institutional biases may be present, and future research is warranted. However, many of the features examined, including the messages, tracker, and air quality index, are used in other pediatric digital health apps [18]. Thus, we expect findings from this study may be informative and relevant to other institutions, caregivers, and children. Despite a diverse racial, ethnic, and socioeconomic sample, most app engagers were White families and well-educated; thus, selection bias may be present. Sampling bias also may exist, even though most eligible participants consented.

Overall, findings from this study show initial relations between family characteristics and app engagement; however, a larger, more diverse study sample is needed to better understand the reasons underlying app engagement. Although we did not find significant correlations between frequent app engagement and participant characteristics, correlational results between app themes and these characteristics suggest they should be considered in future research. Future research should also consider using partial correlations to further examine the relation between app engagement themes and participant characteristics. Other caregiver characteristics, such as being a health care professional and having previously engaged with digital health tools, should be considered, as this may indicate that these caregivers have greater knowledge of asthma and experience with digital health apps and may be more likely to engage with the app. Finally, post hoc analysis revealed responses about app engagement were skewed toward app engagers, as they were more likely to complete the Time 2 survey than nonapp engagers. Despite this skew, most participants completed the Time 2 survey (n=65). Furthermore, post hoc analysis revealed that race and maternal education were not statistically significantly related to completion of the Time 2 survey. Importantly, future digital health studies must work to engage a large, diverse patient population to ensure collecting a representative view of app engagement.

### Conclusions

App engagement continues to demonstrate a digital divide in which children and their caregivers from lower educational and marginalized racial and ethnic backgrounds appear less likely to engage with these tools. Caregiver-reported reasons for not engaging with the app revealed gaps that health care systems need to address to improve the experience and potentially increase engagement of more diverse patient populations. A key finding from this study revealed technological and educational barriers to app engagement. These barriers may be reduced by use of video informational technologies to help better inform caregivers and their children about potential benefits of app engagement and to explain how to use the app. As apps become more ubiquitous in pediatric health care systems, these institutions must strive to ensure that the digital divide does not further exacerbate long-standing equity concerns. This study uncovers some barriers to app engagement and provides some recommendations to assist with reducing gaps in access to and use of digital health technology, which may potentially help improve health and educational outcomes for children.

## Supplementary material

10.2196/69755 Multimedia Appendix 1 App experience survey and supplementary analyses–multiply imputed and post hoc analyses.

## Abbreviations

C-ACT: childhood asthma control test
EHR: electronic health care record
NCH: Nemours Children’s Health
OR: odds ratio
REDCap: Research Electronic Data Capture
