# Supplementary material for: Benefits and Barriers of Caregiver App Engagement for Supporting Diverse Children With Asthma: Mixed Methods Study
Source: JMIR Pediatr Parent. 2025 Dec 4;8:e69755. doi: 10.2196/69755 (PMC12677863; doi:10.2196/69755)
Supplement: Multimedia Appendix 1 [file pediatrics-v8-e69755-s001.docx]

## Section A App Experience Survey

Please read and respond to each question. For questions asking about your child, please answer for your child who has asthma and is currently 5 to 11 years old. For questions about your child’s provider, please answer questions about the provider who your child sees most frequently for his or her asthma care.

1. What month and year was your child first diagnosed with asthma? _________________ (Month and Year)
2. What grade is your child in at school?
   1. Pre-kindergarten
   2. Kindergarten
   3. First grade
   4. Second grade
   5. Third grade
   6. Fourth grade
   7. Fifth grade
   8. Sixth grade
   9. Other (specify):_________________________________________________________
3. How many days over the past year has your child missed school because of asthma?
   1. None
   2. 1-5
   3. 6-10
   4. More than 10
4. Do you think a mobile application (App) would help you and your family manage your child’s asthma?
   1. No
   2. Yes
5. Do you use or have you used the Nemours App? Using the Nemours App means you have logged into the App and used at least one of the features (eg, reminder notifications, asthma tracker, video visit, asthma action plan, messaging system, etc.)
   1. No
   2. Yes
6. Why don’t you use the Nemours App (please select all that apply)?
   1. I didn’t know about it
   2. I’m not good with technology
   3. I’m too busy
   4. I don’t think an App will help manage my child’s asthma
   5. Other (specify): __________________________________________________________________________________________________________________________________________
7. What features on the Nemours App would increase the likelihood that you would use it in the future (please select all that apply)?
   1. Reminder notifications for medication
   2. Educational features (videos, pictures of medications, asthma center, etc.)
   3. Asthma Tracker to monitor asthma control level
   4. Access to Video Visit (virtual medical system to access medical providers online)
   5. Interactive asthma action plan
   6. Notifications about Air Quality Index
   7. Messaging system to communicate with medical providers
   8. Ability to discuss data I entered into the App with my child’s provider
   9. Nothing would increase the likelihood of me or my family using the Nemours App
   10. Other (specify): ____________________________________________________________________________________________________________________________________
8. What barriers have prevented you from using the Nemours App?

________________________________________________________________________________________________________________________________________________

END OF SURVEY FOR NONAPP ENGAGERS

1. How did you find out about the Nemours App (please select all that apply)?
   1. Email from Nemours
   2. A Nemours provider
   3. A family member, friend, or colleague
   4. Other (specify): __________________________________________________________________
2. When did you first log into the Nemours App? __________________ (Month and Year)
3. Who in your home typically enters data into the Nemours App (please select all that apply)?
   1. Parent
   2. Child
   3. Other (specify): __________________________________________________________________
4. What time(s) of day do you use the Nemours App and why (please select all times that apply)?
   1. Morning (please explain):

____________________________________________________________________________________________________________________________________

- 1. Afternoon (please explain):

____________________________________________________________________________________________________________________________________

- 1. Evening (please explain):

____________________________________________________________________________________________________________________________________

1. In general, how often do you use the Nemours App?
   1. Rarely
   2. Once every few months
   3. Once a month
   4. A few times a month
   5. Once a week
   6. A few times a week
   7. Everyday
2. What feature(s) do you use on the Nemours App and how frequently do you use them (please select all that apply)?
   1. Reminder notifications for medication
      1. Rarely
      2. Once every few months
      3. Once a month
      4. A few times a month
      5. Once a week
      6. A few times a week
      7. Everyday
   2. Educational features (videos, pictures of medications, asthma center, etc.)
      1. Rarely
      2. Once every few months
      3. Once a month
      4. A few times a month
      5. Once a week
      6. A few times a week
      7. Everyday
   3. Asthma Tracker to monitor asthma control level
      1. Rarely
      2. Once every few months
      3. Once a month
      4. A few times a month
      5. Once a week
      6. A few times a week
      7. Everyday
   4. Video Visit (telehealth virtual medical system to access medical providers online)
      1. Rarely
      2. Once every few months
      3. Once a month
      4. A few times a month
      5. Once a week
      6. A few times a week
      7. Everyday
   5. Interactive asthma action plan
      1. Rarely
      2. Once every few months
      3. Once a month
      4. A few times a month
      5. Once a week
      6. A few times a week
      7. Everyday
   6. Notifications about Air Quality Index
      1. Rarely
      2. Once every few months
      3. Once a month
      4. A few times a month
      5. Once a week
      6. A few times a week
      7. Everyday
   7. Messaging system to communicate with medical providers
      1. Rarely
      2. Once every few months
      3. Once a month
      4. A few times a month
      5. Once a week
      6. A few times a week
      7. Everyday
   8. Discussing data I entered into the Nemours App with my child’s provider
      1. Rarely
      2. Once every few months
      3. Once a month
      4. A few times a month
      5. Once a week
      6. A few times a week
      7. Everyday
   9. Other (specify the feature you use and describe how often you use this feature): ____________________________________________________________________________________________________________________________________
3. What do you like about the Nemours App (describe)? ________________________________________________________________________________________________________________________________________________
4. What don’t you like about the Nemours App (describe)?

________________________________________________________________________________________________________________________________________________________________________________________________________________________

1. What barriers, if any, have prevented you from using the Nemours App?

________________________________________________________________________________________________________________________________________________

1. Do you think using the Nemours App has improved your child’s medication use?
   1. No
   2. Yes

15a. What features have you used that you think have helped improve medication use (please select all that apply)?

- 1. Reminder notifications for medication
  2. Educational features (videos, pictures of medications, asthma center, etc.)
  3. Asthma Tracker to monitor asthma control level
  4. Video Visit (telehealth virtual medical system to access medical providers online)
  5. Interactive asthma action plan
  6. Notifications about Air Quality Index
  7. Messaging system to communicate with medical providers
  8. Discussing data I entered into the App with my child’s provider
  9. Other (specify): ____________________________________________________________________________________________________________________________________

1. Are there any additional barriers to taking medication as prescribed that the Nemours App cannot address (please select all that apply)?
   1. Insurance does not cover medications needed
   2. Child refuses to take medication as prescribed
   3. Other (specify): ____________________________________________________________________________________________________________________________________
2. Do you think using the Nemours App has helped you better manage your child’s asthma symptoms?
   1. No
   2. Yes

17a. What features have you used that have helped you to better manage your child’s asthma symptoms (please select all that apply)?

- 1. Reminder notifications for medication
  2. Educational features (videos, pictures of medications, asthma center, etc.)
  3. Asthma Tracker to monitor asthma control level
  4. Video Visit (telehealth virtual medical system to access medical providers online)
  5. Interactive asthma action plan
  6. Notifications about Air Quality Index
  7. Messaging system to communicate with medical providers
  8. Discussing data I entered into the App with my child’s provider
  9. Other (specify): ____________________________________________________________________________________________________________________________________

1. Do you think using the Nemours App has improved communication between your family and your child’s provider?
   1. No
   2. Yes

18a. What features have you used that you think have helped improve communication between your family and your child’s provider (please select all that apply)?

- 1. Reminder notifications for medication
  2. Educational features (videos, pictures of medications, asthma center, etc.)
  3. Asthma Tracker to monitor asthma control level
  4. Video Visit (telehealth virtual medical system to access medical providers online)
  5. Interactive asthma action plan
  6. Notifications about Air Quality Index
  7. Messaging system to communicate with medical providers
  8. Discussing data I entered into the App with my child’s provider
  9. Other (specify): ____________________________________________________________________________________________________________________________________

1. Have you used the Asthma Tracker to track your child’s symptoms?
   1. No
   2. Yes

19a. Have you discussed your asthma tracking entries with your child’s provider?

1. No (please explain why not):

______________________________________________________________________________________________________________________________________________________________________________________________________

1. Yes (please explain why):

______________________________________________________________________________________________________________________________________________________________________________________________________

1. Have you discussed any other features of the Nemours App with your child’s provider or used the App to communicate with your child’s provider?
   1. No
   2. Yes

20a. What features have you discussed with your child’s provider or used to communicate with your child’s provider (please select all that apply)?

1. Reminder notifications for medication
2. Educational features (videos, pictures of medications, asthma center, etc.).
3. Asthma Tracker to monitor asthma control level
4. Video Visit (telehealth virtual medical system to access medical providers online)
5. Interactive asthma action plan
6. Notifications about Air Quality Index
7. Messaging system to communicate with medical providers
8. Discussing data I entered into the App with my child’s provider
9. Other (specify): ____________________________________________________________________________________________________________________________________
10. Do you think using the Nemours App has improved your knowledge of your child’s asthma?
    1. No
    2. Yes

21a. What features have you used that you think have helped improve your knowledge of your child’s asthma (please select all that apply)?

1. Reminder notifications for medication
2. Educational features (videos, pictures of medications, asthma center, etc.)
3. Asthma Tracker to monitor asthma control level
4. Video Visit (telehealth virtual medical system to access medical providers online)
5. Interactive asthma action plan
6. Notifications about Air Quality Index
7. Messaging system to communicate with medical providers
8. Discussing data I entered into the App with my child’s provider
9. Other (specify): ____________________________________________________________________________________________________________________________________
10. Have you learned anything new about your child’s asthma since using the Nemours App?
    1. No
    2. Yes (please specify):

______________________________________________________________________________________________________________________________________________________________________________________________________

22a. What features have you used that you think have helped you learn something new about your child’s asthma (please select all that apply)?

1. Reminder notifications for medication
2. Educational features (videos, pictures of medications, asthma center, etc.)
3. Asthma Tracker to monitor asthma control level
4. Video Visit (telehealth virtual medical system to access medical providers online)
5. Interactive asthma action plan
6. Notifications about Air Quality Index
7. Messaging system to communicate with medical providers
8. Discussing data I entered into the App with my child’s provider
9. Other (specify): ____________________________________________________________________________________________________________________________________
10. Have you used the Video Visit feature?
    1. No
    2. Yes

23a. If yes, what did you use the Video Visit for and did the appointment resolve the issue?

________________________________________________________________________________________________________________________________________________________________________________________________________________________

1. Have you experienced any difficulties using the Nemours App?
   1. No
   2. Yes (please explain):

______________________________________________________________________________________________________________________________________________________________________________________________________

1. Is there anything else you would like to share about your experience using the Nemours App?

______________________________________________________________________________________________________________________________________________________________________________________________________

__________________________________________________________________

END OF SURVEY FOR APP ENGAGERS

## Section B Multiply Imputed Analysis––Predicting App Engagement From Demographic Survey and Health Care Records

The full logistic regression model was statistically significant, (χ^2^,3=36.0; P<.001), and well specified, as the Hosmer-Lemeshow Test was not statistically significant (P=.90). The model explained approximately 49% of the variance for app engagement (Nagelkerke R^2^=.49) and correctly classified 80% of the cases. Caregivers of White children were nearly 5.5 times more likely to engage with the app than caregivers of children of other races when controlling for maternal education and child asthma control level (OR [odds ratio] 5.35, 95% CI 1.61-17.71; P=.01). Caregivers with a college degree were over 4.5 times more likely to engage with the app than caregivers with a high school diploma when controlling for child race and asthma control level (OR 4.66, 95% CI 1.40-15.53; P=.01). Caregivers of children with uncontrolled asthma were almost 22 times more likely to engage with the app than caregivers of children with controlled asthma when controlling for maternal education and child race (OR 21.74, 95% CI 3.97-119.05; P<.001).

## Section C Multiply Imputed Analysis––Relation Between App Engagement and Caregiver-Reported School Absences

Chi-square analysis revealed that app engagement was statistically significantly related to the school absence difference score (0=no change in absences, 1=absences reduced by 1 category, 2=absences reduced by 2 categories, 3=absences reduced by 3 categories) between Time 1 and Time 2 (χ^2^,3=17.0; P<.001). Post hoc analyses using Bonferroni correction to correct for Type 1 error (P=.01) revealed absences reduced by 3 categories was statistically significantly related to app engagement (χ^2^,3=15.8; P=.01), suggesting that caregivers who did not use the app had a child with significantly fewer absences during the study. Due to discrepancies in the caregiver-reported absentee data between Time 1 and Time 2, we caution these results may be uninterpretable and warrant further research.

## Section D Post Hoc Analyses to Examine Caregiver-Reported School Absences

Post hoc chi-square analyses indicated a significant relation between school absences and race (P=.04), asthma control (P=.01), and app engagement (P<.001), but not maternal education (P=.18). Due to discrepancies in the caregiver-reported absentee data between Time 1 and Time 2, we caution these results may be uninterpretable, warranting further research.
